# Diffractive optics for combined spatial- and mode- division demultiplexing of optical vortices: design, fabrication and optical characterization

**DOI:** 10.1038/srep24760

**Published:** 2016-04-20

**Authors:** Gianluca Ruffato, Michele Massari, Filippo Romanato

**Affiliations:** 1 Department of Physics and Astronomy ‘G. Galilei’, University of Padova, via Marzolo 8, 35131 Padova, Italy; 2 LaNN, Laboratory for Nanofabrication of Nanodevices, Corso Stati Uniti 4, 35127 Padova, Italy; 3 CNR-INFM TASC IOM National Laboratory, S.S. 14 Km 163.5, 34012 Basovizza, Trieste, Italy

## Abstract

During the last decade, the orbital angular momentum (OAM) of light has attracted growing interest as a new degree of freedom for signal channel multiplexing in order to increase the information transmission capacity in today’s optical networks. Here we present the design, fabrication and characterization of phase-only diffractive optical elements (DOE) performing mode-division (de)multiplexing (MDM) and spatial-division (de)multiplexing (SDM) at the same time. Samples have been fabricated with high-resolution electron-beam lithography patterning a polymethylmethacrylate (PMMA) resist layer spun over a glass substrate. Different DOE designs are presented for the sorting of optical vortices differing in either OAM content or beam size in the optical regime, with different steering geometries in far-field. These novel DOE designs appear promising for telecom applications both in free-space and in multi-core fibers propagation.

Since the seminal paper of Allen *et al*. in 1992^1^, the orbital angular momentum (OAM) of light has known an increasing attention with applications in a wide range of fields^2^,^3^,^4^ as: particle trapping^5^ and tweezing^6^, phase contrast microscopy^7^, stimulated emission depletion (STED) microscopy^8^, astronomical coronagraphy^9^, quantum-key distribution^10^ and telecommunications^11^,^12^. In the last field, the exploitation of this novel degree of freedom in order to enhance information-carrying capacity and spectral efficiency of today’s networks has provided a promising solution to tackle the worldwide overwhelming appetite of bandwidth both in the radio and optical regimes. On the other hand, a few crucial points represent still open technological issues that require further optimization before commercial applications in the optical domain. Among these we include the insertion of OAM modes in the optical fiber, the further optimization for their long distance propagation and, finally, the (de)multiplexing technique exploited for OAM-mode sorting.

As far as demultiplexing is concerned, several methods have been presented and characterized in order to separate a set of multiplexed beams with different OAM contributions: interferometric methods^13^, optical transformations^14^,^15^,^16^, time-division techniques^17^, integrated silicon photonics^18^, coherent detection^19^, diffractive optics^20^,^21^,^22^,^23^. With respect to other techniques, diffractive optical elements (DOE) appear to be the most suitable choice for the realization of passive and lossless, compact and cheap optical devices for integrated (de)multiplexing applications with high flexibility in the output pattern geometry^24^. Diffractive analysers have been widely presented and exploited in literature for the analysis of OAM beam superposition. The far-field of such optical elements exhibits bright peaks at prescribed positions, whose intensity is proportional to the contributions of the corresponding OAM channels in the incident beam.

An OAM-carrying beam is characterized by an azimuthally varying phase term 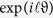, being 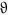 the angular coordinate on a plane perpendicular to the optical axis and *ℓ* the OAM content per photon in units of *h*/2*π*. A peculiar characteristic is the presence of a central dark singularity surrounded by a ring distribution of field intensity. This feature allows the realization of diffractive optical elements merely acting on the zone with non-null incident field. Then the non-illuminated regions can be exploited for other purposes. For instance, diffractive optical elements composed of concentric zones acting on OAM beams with different radii, i.e. different *ℓ* values, have been designed for space diffraction compensation^25^ or add/drop operations between different OAM channels^26^. On the other hand, stable propagation of OAM modes along ring fibers has highlighted the need to excite and manipulate annular intensity distributions with fixed radius and width, regardless of the OAM content^27^,^28^. However, conventional OAM beams are limited since their ring diameter increases with the topological charge *ℓ*. For instance, the maximum intensity radius increases linearly with *ℓ* for Kummer beams and as 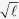 for Laguerre-Gaussian beams^29^. This property may create problems when coupling multiple OAM beams into a fiber with fixed annular index profile or under manipulation with finite-size optical elements. To overcome this limitation, Ostrovsky and coworkers first introduced the concept of “perfect vortex” proposing OAM beams whose ring-diameter and ring-width are both independent of the topological charge^30^. Therefore these beams allow the transportation of a helical phase-front, together with the confinement of the electromagnetic field within a ring of controlled radius and width. The corresponding annular field profile *E*_*ℓ*_ at a fixed propagation distance and for a specific OAM *ℓ* can be approximated by^31^:


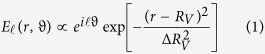


where (*r, ϑ*) are polar coordinates, *R*_*V*_ and Δ*R*_*V*_ define radius and width of the intensity annulus respectively. Different methods were presented in order to generate and tailor perfect vortices. In Ostrovsky’s work the method relies on the implementation of a Fourier transforming optical system with a phase pattern created by a programmable spatial light modulator (SLM)^32^. Other groups exploited a different method by illuminating a vortex phase mask by means of an annular beam (created for instance using an axicon)^31^,^33^,^34^. More recently a new technique to form a perfect vortex beam with controllable ring radius using the Fourier transform property of a Bessel beam has been presented^35^. In this work, perfect vortices are generated illuminating with a Gaussian beam a phase pattern loaded on a spatial light modulator that implements the combination of a spiral term and of an axicon contribution, as exploited elsewhere^28^. A lens is applied for annulus collimation. The radius of the generated annular beam is determined by the axicon parameter along with the propagation distance past the axicon, whereas the annulus width is inversely proportional to the incident Gaussian beam waist.

Here we introduce the exploitation of diffractive optics for the demultiplexing of perfect vortices. Since the width of the intensity ring can be much narrower than in common OAM beams, there is a remarkable reduction of the illuminated area, and therefore a non-trivial saving in lithographic time and costs. Moreover, taking advantage of the radial confinement of these beams, we designed more complex diffractive optical elements for the sorting of coaxial vortices illuminating non-overlapping zones of the optical devices. Samples have been fabricated with high-resolution electron-beam lithography (EBL) and tested on an optical table at the wavelength *λ* = 632.8 nm. By properly controlling the impinging beam size and in particular the far-field spot pattern, the described optical elements allow performing OAM-mode division demultiplexing (OAM-MDM) and spatial division multiplexing (SDM) with the same optical platform. Moreover, the directions of the demultiplexed beams in far-field can be arbitrarily controlled by properly designing the DOE phase pattern.

## Results

### DOE concept and design

**Figure 1:**
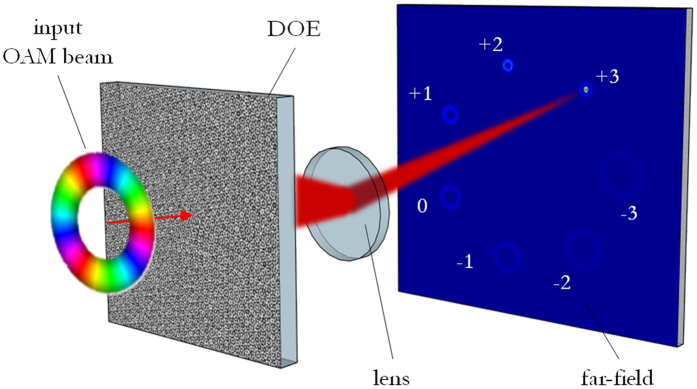
Concept and principle of OAM-mode division demultiplexing (OAM-MDM) with diffractive optics. In the specific case: input OAM beam with *ℓ* = +3 impinges upon a DOE for OAM-MDM of 7 channels in the range *ℓ* = −3, …, +3. A bright spot appears in far-field in correspondence of the position for *ℓ* = +3.

The phase pattern of a diffractive optics intended for expanding the incident light field into different diffraction orders (Fig. 1) is given by the linear combination of *n* angular harmonics {*ψ*_*i*_* = *exp(*iℓϑ*)} as it follows^20^:


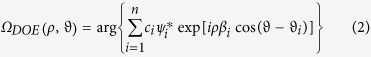


where {(*β*_*i*_, *ϑ*_*i*_)} are the *n* vectors of carrier spatial frequencies in polar coordinates and *c*_*i*_ are complex coefficients whose modulus is given arbitrarily and the arguments are free parameters of the task, fitted in such a manner that equation (2) becomes an exact equality. The coefficients are given by the following relation:


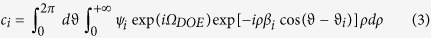


A custom code implemented in MATLAB is used to calculate the phase pattern for given sets of OAM values {*ℓ*_*i*_} and carrier spatial frequencies {(*β*_*i*_, *ϑ*_*i*_)}. The implemented algorithm is based on a successive computation of the sum in equation (2) and integrals in equation (3), using the fast Fourier transform algorithm and considering definite limitations. At the *p*th iteration, the coefficients *c*_*i*_^(*p*)^ are replaced by *c*_*i*_^(*p*)*^ as it follows^36^:


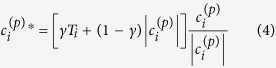


where *T*_*i*_ > 0 are pre-set numbers characterizing the response of every channel (usually *T*_*i*_ = 1, ∀ *i*), and 0 < *γ* ≤ 2 is a relaxation coefficient controlling the algorithm convergence. Then this estimate is put in equation (2) and the iteration is repeated until convergence. At any step, phase quantization is applied, since the continuous complex spectrum obtained in the DOE plane is assumed to be finally fabricated by a lithographic process that is usually capable of producing structures with only discretely-valued functions of transmittance, due to limited dose-accuracy dependent *z*-resolution. Hence, the phase interval [0, 2π] is discretized into a finite number *N* of phase levels {2π*i*/*N*}, *i* = 0, …, *N*-1.

**Figure 2:**
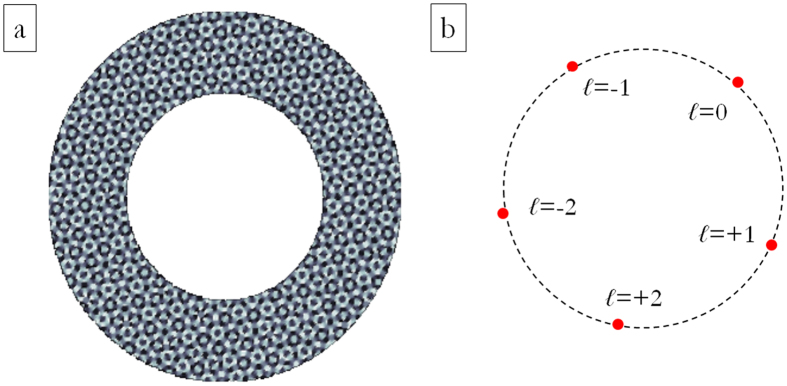
(**a**) Phase pattern of DOE performing OAM-MDM of optical vortices in the range {−2, −1, 0, +1, +2}, 8 phase levels: 0, π/4, π/2, 3π/4, π, 5π/4, 3π/2, 7π/4. Inner ring: 300 μm, outer ring 500 μm. Numerical calculation with custom MATLAB code. (**b**) Scheme of channels constellation in the far-field.

For the demultiplexing of perfect vortices, it is sufficient to consider only the phase-pattern zone where the incident field is non-null. Figure 2(a) exhibits the calculated phase pattern for the sorting of perfect vortices with OAM values in the range {−2, −1, 0, +1, +2} and electromagnetic field confined within a ring with inner and outer radii respectively of 350 μm and 500 μm. At the back-focal plane of a lens with focal length *f*, the signal spots are located along a circle with radius *r* = *βf*/*k* and equally-spaced angular positions with a step of 2π/5, being *k* = 2π/*λ* the wavevector in air. With such a choice, we prevent any channel from overlapping with the zero-order term, which is usually a noise carrier because of unavoidable defects in sample fabrication.

The previous configuration can be replicated in the form of a multi-ring DOE combining OAM-MDM and SDM demultiplexing techniques. The total phase pattern Ω_DOE_ can be obtained by the composition of *s* different concentric phase patterns Ω^*j*^_DOE_, according to:


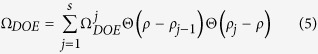


being Θ the Heaviside function and *ρ*_*j*−1_ and *ρ*_*j*_ the internal and external radii of the *j*th DOE pattern Ω^*j*^_DOE_ given by equation (2), with carrier spatial frequencies {(*β*_*i*_^*j*^, *ϑ*_*i*_^*j*^)}, *j* = 1, …*s, i* = 1, …*n*. Positions {(*r*_*i*_^*j*^, *φ*_*I*_^*j*^)} of the signal spots in far-field will be given by:


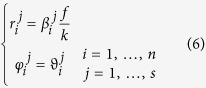


being *f* the focal length of the lens exploited for far-field reconstruction in *f-f* configuration.

**Figure 3:**
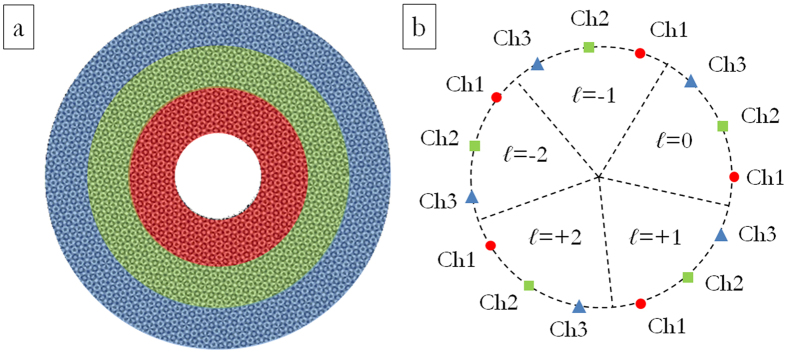
(**a**) Phase pattern of DOE for combined OAM-MDM and SDM of optical vortices. OAM values in the range {−2, −1, 0, +1, +2}, 8 phase levels: 0, π/4, π/2, 3π/4, π, 5π/4, 3π/2, 7π/4. Numerical calculation with custom MATLAB code. (**b**) Scheme of channels constellation in the DOE far-field. Marker colours and forms refer to the three different radial channels (inner ring: red circles–central ring: green squares–outer ring: blue triangles). Number of total OAM channels: 15.

At first we limited our choice to *s* = 3 and *n* = 5, with *ℓ* values in the set {−2, −1, 0, +1, +2} for a total of 15 OAM channels. In addition, we chose the carrier spatial frequencies so that the far-field peaks were arranged along a circle of constant radius *r* and equally-spaced angular positions (see Fig. 3(b)), specified as it follows:


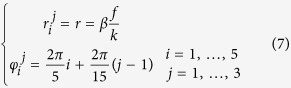


In practice, we firstly calculated the phase pattern corresponding to a DOE performing demultiplexing over 5 OAM beams in the set {*ℓ*_*i*_} and carrier frequencies given by equation (7) with *j* = 1. Then the two other DOE rings were obtained by rotating the previous pattern, sequentially, by 2π/15. Therefore the three phase patterns were cropped according to the sizes of the impinging optical vortices and juxtaposed one another in order to build up the total DOE pattern.

**Figure 4:**
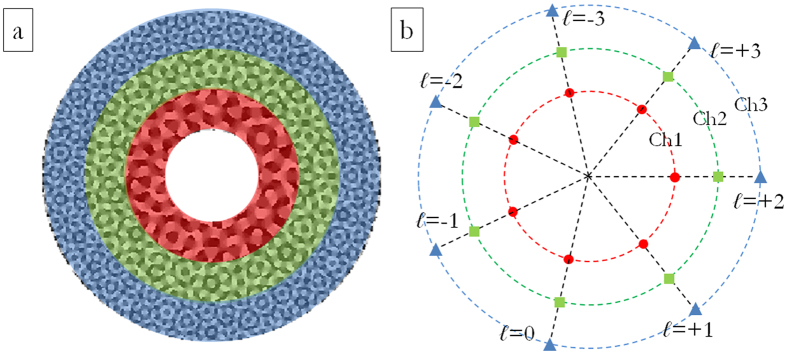
(**a**) Phase pattern of DOE for combined OAM-MDM and SDM of perfect vortices. OAM values in the range {−3, −2, −1, 0, +1, +2, +3}, 8 phase levels: 0, π/4, π/2, 3π/4, π, 5π/4, 3π/2, 7π/4. Numerical calculation with custom MATLAB code. (**b**) Scheme of channels constellation in the DOE far-field. Marker colours and forms refer to the three different radial channels (inner ring: red circles–central ring: green squares–outer ring: blue triangles). Number of total OAM channels: 21.

For the sake of completeness, we show also the case when an increased set of OAM values and both the azimuthal and the radial degrees of freedom are exploited in order to arrange demultiplexed channels in far-field for combined OAM-MDM and SDM. This second DOE pattern is designed in order to have *s* = 3 and *ℓ* values in the set {−3, −2, −1, 0, +1, +2, +3}, i.e. *n* = 7, for a total of 21 OAM channels. In this case, the carrier spatial frequencies were chosen so that the far-field peaks were arranged on three different circles, corresponding to the three different beam sizes, and equally-spaced angular positions (see Fig. 4(a)), as it follows:


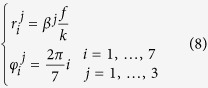


### Fabrication

**Figure 5:**
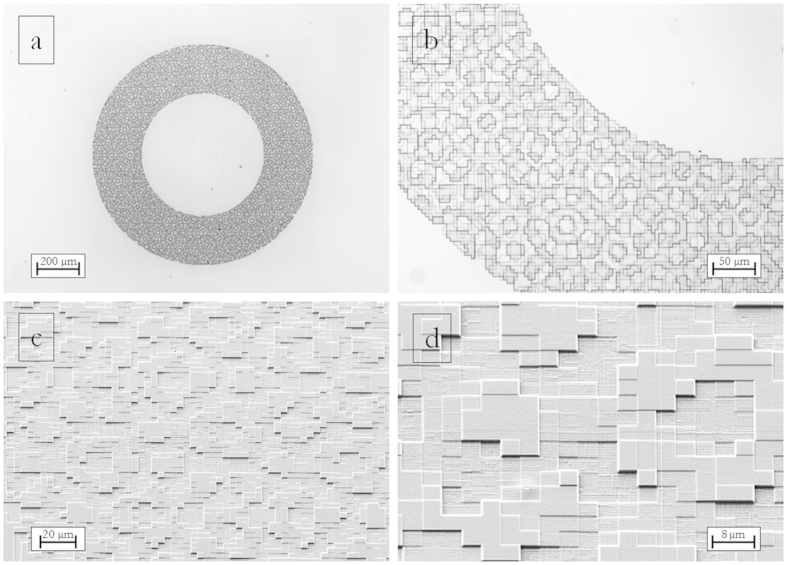
Optical microscopy (**a,b**) and scanning electron microscopy (**c,d**) of a PMMA phase-only DOE for mode-division demultiplexing of optical vortices with OAM values in the range {−3, −2, −1, 0, +1, +2, +3}. Inner radius 300 μm, outer radius 500 μm. Pixel size: 4 × 4 μm^2^. Working wavelength *λ* = 632.8 nm. 8 step levels, nominal heights (according to equation (9)): *d*_1_ = 0 nm, *d*_2_ = 161.8 nm, *d*_3_ = 323.5 nm, *d*_4_ = 485.3 nm, *d*_5_ = 647.0 nm, *d*_6_ = 808.8 nm, *d*_7_ = 970.6 nm, *d*_8_ = 1132.3 nm.

**Figure 6:**
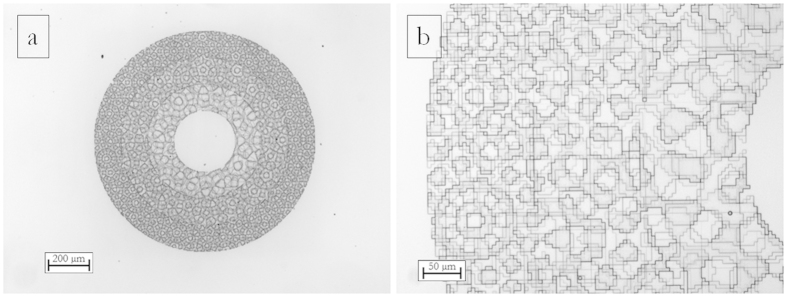
Optical microscopy of a PMMA phase-only DOE for combined OAM-MDM and SDM of optical vortices with OAM values in the set {−3, −2, −1, 0, +1, +2, +3} and three ranges of beam size, according to the scheme in Fig. 4. Inner radius 140 μm, outer radius 500 μm. Width of each annulus: 120 μm. Pixel size: 4 × 4 μm^2^. Working wavelength *λ* = 632.8 nm. 8 phase levels.

Phase-only diffractive optical elements are fabricated as surface-relief patterns of pixels. This 3-D structure can be realized by shaping a layer of transparent material, imposing a direct proportionality between the thickness of the material and the local phase delay. Electron beam lithography (EBL) is the ideal technique in order to fabricate 3D profiles with the required high resolution^37^,^38^,^39^. By modulating the local dose distribution, a different dissolution rate is induced in the exposed polymer, giving rise to different resist thicknesses after the development process. In this work the DOE patterns were written on a polymethylmethacrylate (PMMA) resist layer with a JBX-6300FS JEOL EBL machine, 5 nm resolution, working at 100 keV with a current of 100 pA. The substrate used for fabrication is glass, coated with an ITO layer with low surface resistivity (8–12 Ω) in order to ensure a good discharge of the sample during electron beam lithography. After the exposure, the resist is developed in a temperature-controlled developer bath for 60 s. The quality of the fabricated structures has been assessed using Optical Microscopy (Fig. 5 (a,b) and 6), Scanning Electron Microscopy (SEM) (Fig. 5 (c,d)) and Atomic Force Microscopy (AFM) (see Supplementary Figure 1).

At the experimental wavelength of the laser (*λ* = 632.8 nm), PMMA refractive index results *n*_PMMA_ = 1.489 from spectroscopic ellipsometry analysis (J.A. Woollam VASE, 0.3 nm spectral resolution, 0.005° angular resolution). The height *d*_*k*_ of the pixels belonging to the *k*th layer is given, for normal incidence in air, by:


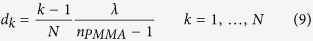


being *N* the total number of phase levels. The DOE pixels belong to a 250 × 250 square matrix with *N* = 8 phase levels. Each pixel is 4 × 4 μm^2^, therefore the total area of the sample is 1 mm^2^. Inserting the given laser wavelength and PMMA refractive index in equation (9) we get: *d*_1_ = 0 nm, *d*_2_ = 161.8 nm, *d*_3_ = 323.5 nm, *d*_4_ = 485.3 nm, *d*_5_ = 647.0 nm, *d*_6_ = 808.8 nm, *d*_7_ = 970.6 nm, *d*_8_ = 1132.3 nm. Experimental height values have been compared with the nominal ones exhibiting a remarkable accordance within the experimental errors, estimated by considering surface roughness. Roughness root-mean-square (RMS) increases from 4 nm to 32 nm, for the lowest and highest depth level respectively (see Supplementary Figure 1).

For combined OAM-MDM and SDM analysis, the DOE pattern is the composition of 3 annuli with edges: *ρ*_0_ = 140 μm, *ρ*_1_ = 260 μm, *ρ*_2_ = 380 μm, *ρ*_3_ = 500 μm (see Fig. 6), designed accordingly to the schemes in Figs 3 and 4.

### Optical response

**Figure 7:**
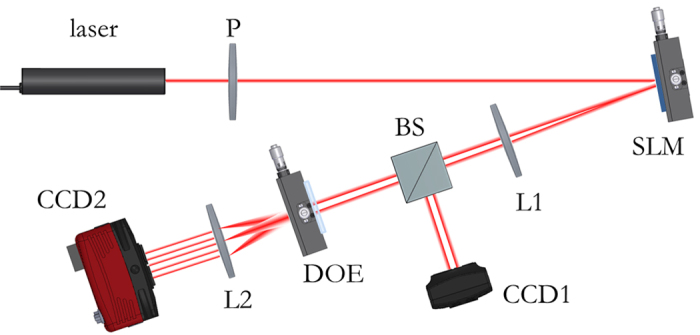
Scheme of the optical characterization setup. Laser source (*λ* = 632.8 nm), linear polarizer (P), LCoS spatial light modulator (SLM), first lens (L1), beam splitter (BS), diffractive optical element (DOE) mounted on XY micrometric translator, second lens (L2), cameras for analysis of the DOE input and output signals (CCD1, CCD2).

The characterization setup was mounted on an optical table (see scheme in Fig. 7). The Gaussian beam (*λ* = 632.8 nm, beam waist *w*_0_ = 240 μm) emitted by a HeNe laser source illuminates the display of a reflective liquid-crystal-on-silicon (LCoS) spatial light modulator (PLUTO-NIR-010-A, Holoeye) for perfect vortex generation. Then the beam is collimated with a first lens of focal length *f*_1_ = 25 cm and a beam-splitter is placed in order to allow analyzing the field profile and its OAM content at the same time. The beam illuminates the DOE sample, fixed on a *XY* translation mount with micrometric drives and the far-field is collected at the back-focal plane of a lens of focal length *f*_2_ = 10 cm.

The SLM implements a phase mask Ω_SLM_ that combines axicon and spiral phase functions with a quadratic-phase term for curvature correction:


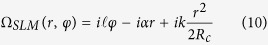


where *α* is the axicon parameter, *k* is the wavevector in air, and *R*_*c*_ is the curvature radius of the incident beam. If the SLM is located at a distance *z* from the laser source, the curvature term can be estimated as *R*_*c*_ = (*z*^2^ + *z*_R_^2^)/*z*, being *z*_R_ = π*w*_0_^2^/*λ* the Rayleigh range. At the focal plane of the first lens, the axicon function forms a ring having the diameter *R*_V_ varying with the axicon parameter *α* and the focal length *f*_1_ according to:


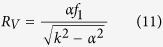


The spiral phase term gives the vortex nature with topological charge *ℓ* to the ring beam. The ring-width can be controlled by changing the incident beam radius *w*, according to Δ*R*_V_ = 2*f*_1_/(*k w*), where *w* = *w*_0_[1 + (*z*/*z*_*R*_)^2^]^1/2^. In our case of interest, the ring width results around 2·Δ*R*_V_ = 55 μm. Three axicon parameters, *α*_1_ = 0.008 μm^−1^, *α*_2_ = 0.012 μm^−1^, *α*_3_ = 0.0175 μm^−1^, were alternatively chosen in order to generate perfect vortices with radii around respectively *R*_1_ = 200 μm, *R*_2_ = 320 μm, *R*_3_ = 440 μm, illuminating the corresponding DOE annulus without overlapping.

**Figure 8:**
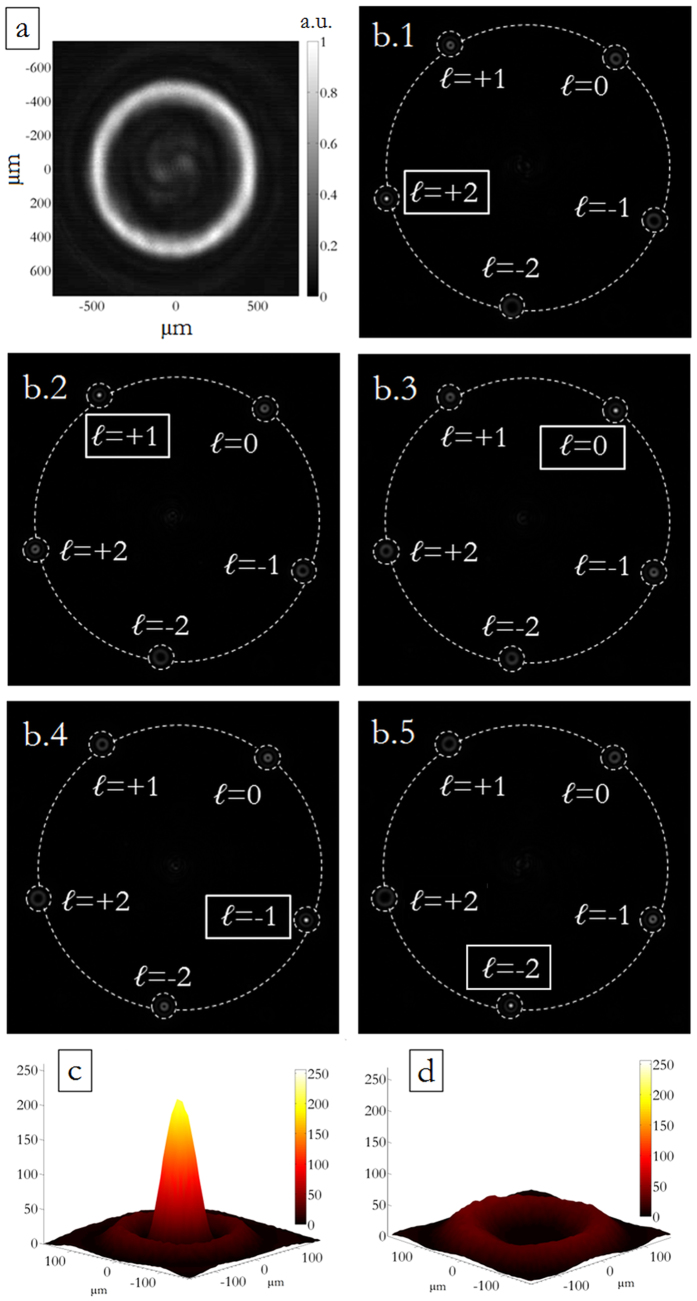
(**a**) Measured intensity of the incident perfect vortex, in the specific case with *ℓ* = −2, on a plane perpendicular to the propagation direction. (**b**.1–5) Experimental far-field on the CCD camera of a DOE performing OAM-MDM in the range {−2, −1, 0, +1, +2}, according to the scheme in Fig. 2. The far-field exhibits a bright spot in correspondence of the corresponding input OAM (**c**), otherwise no peak is present (**d**).

**Figure 9:**
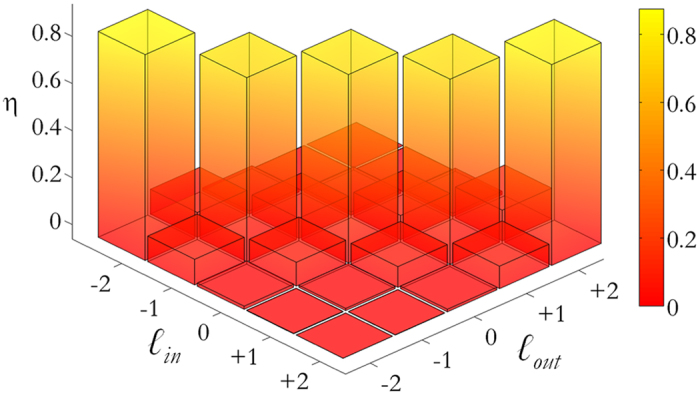
Phase-only DOE for OAM-MDM in the range {−2, −1, 0, +1, +2}, according to the scheme in Fig. 2. Total intensities in all detector regions for perfect vortex input modes, for the experimental data shown in Fig. 8. For each channel, the detection regions have the same size and are chosen so that they cover the intensity peak area. Intensities are normalized to the total collected energy.

Depending on the designed phase pattern, the final output consists of an array of spots, whose brightness is proportional to the corresponding OAM contribution to the input beam. For each input OAM signal, energy is collected in far-field over a well-defined matrix of detectors by measuring light intensity at specific points on the CCD image, in correspondence of the different diffraction orders specified by equation (6). In Fig. 8 normalized intensities are reported for a DOE performing OAM-MDM in the range {−2, −1, 0, +1, +2}. The plot exhibits a well-defined channel response, with efficiencies between 77% and 86% (Fig. 9). The cross-talk *XT* of the channel corresponding to a selected value ℓ = ℓ* is defined as:


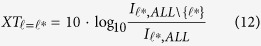


where *I*_ℓ*,ALL_ is the signal in correspondence of channel ℓ* when all input OAM signals in the set {*ℓ*_*i*_} are on, ℓ* included, while *I*_*ALL\*(ℓ*)_ is the signal at channel ℓ* when the input channel ℓ* is off. For the given data set, cross-talk values result between −9.1 and −6.7 dB.

**Figure 10:**
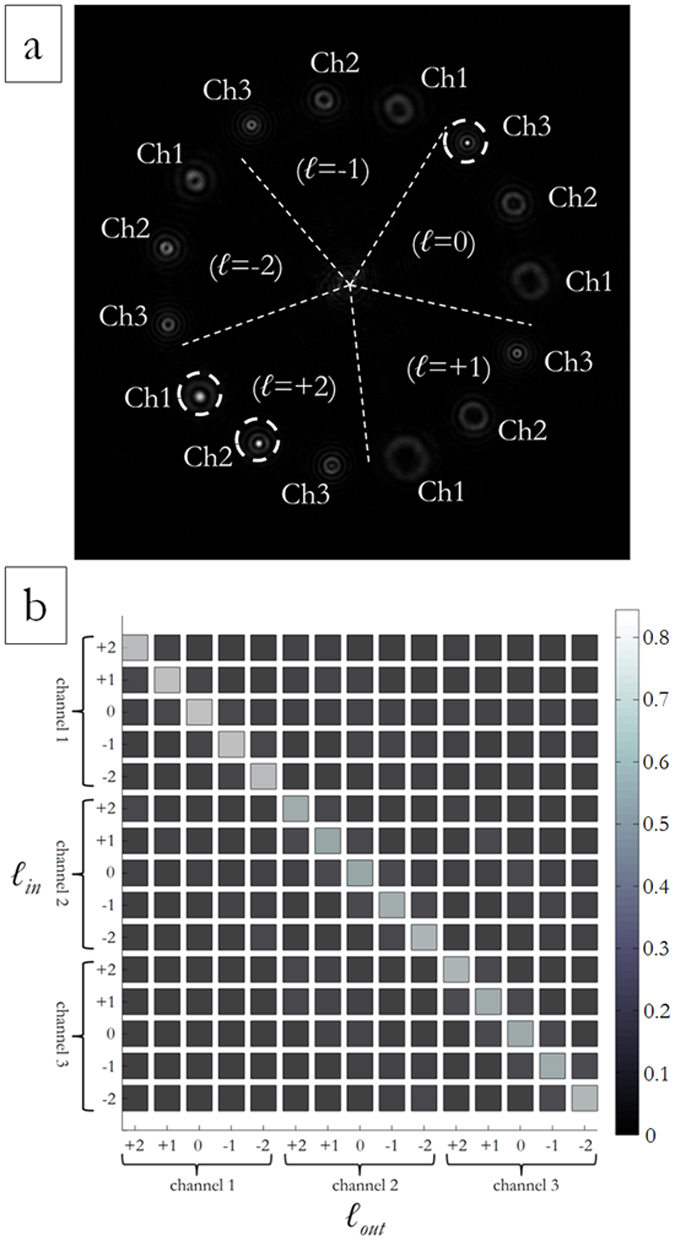
Experimental optical response for a DOE performing combined OAM-MDM and SDM according to the scheme in Fig. 3. (**a**) Experimental output for input OAM_+2,ch1_, OAM_+2,ch2_ and OAM_0,ch3_. (**b**) Total intensities in all detector regions for perfect vortex input modes, experimental data. For each channel, detection regions have the same size and are chosen so that they cover the intensity peak area. Intensities are normalized to the total collected energy.

The same analysis has been carried out for DOEs performing combined OAM-MDM and SDM according to the schemes in Figs 3 and 4. Figure 10(a) exhibits the total far-field for three perfect vortices impinging on a DOE performing demultiplexing of three beam sizes with OAM values in the range {−2, −1, 0, +1, +2} according to the scheme in Fig. 3: *ℓ* = +2 on the first DOE ring (OAM_+2,ch1_), *ℓ* = +2 on the second DOE ring (OAM_+2,ch2_) and *ℓ* = 0 on the third DOE ring (OAM_0,ch3_).

**Figure 11:**
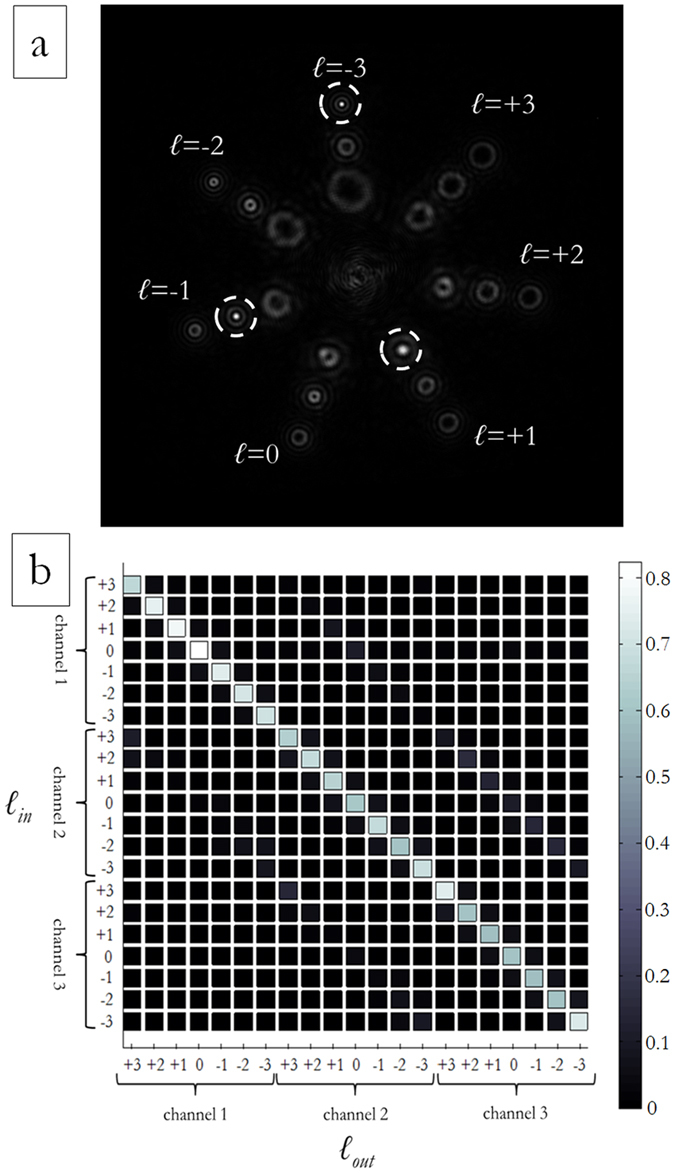
Experimental optical response for a DOE performing combined OAM-MDM and SDM according to the scheme in Fig. 4. (**a**) Experimental output for input OAM_+1,ch1_, OAM_−1,ch2_ and OAM_−3,ch3_. (**b**) Total intensities in all detector regions for perfect vortex input modes, experimental data. For each channel, detection regions have the same size and are chosen such that they cover the intensity peak area. Intensities are normalized to the total collected energy.

Figure 11(a) exhibits the total far-field for three perfect vortices impinging on a DOE performing demultiplexing of three beam sizes with OAM values in the range {−3, −2, −1, 0, +1, +2, +3} according to the scheme in Fig. 4: *ℓ* = +1 on the first DOE ring (OAM_+1,ch1_), *ℓ* = −1 on the second DOE ring (OAM_−1,ch2_) and *ℓ* = −3 on the third DOE ring (OAM_−3,ch3_). In Figs 10(b) and 11(b), the total optical response is shown for both DOE samples. Channel cross-talk could be further reduced by decreasing vortex-ring widths.

## Discussion

The designed phase masks successfully enable OAM-MDM and SDM on the same optical element, while the demultiplexed OAM beams are distributed over the expected geometric patterns in far-field. Therefore we demonstrated the possibility of demultiplexing multiple-size optical vortices carrying different topological charges by means of the same DOE platform. This diffractive optics design is promising in applications where spatial-division multiplexing and OAM-mode division multiplexing are combined for further improvement of the information transmission capacity, in terms of bandwidth, of the optical link, both for free-space transmission and optical fibers applications. For instance, these devices could find applications with novel single-core or coaxial multi-core ring fibers. Moreover, the compatibility of this sorting technique with polarization-division multiplexing (PDM) allows doubling the number of the available OAM channels. A strong point of diffractive optics for MDM is the high flexibility in tailoring the demultiplexed beams pattern. Here we showed the possibility of exploiting both radial and angular degrees of freedom by presenting two DOE samples with different far-field patterns. The first one enables OAM-MDM in the range {−2, −1, 0, +1, +2} and three different beam sizes, for a total of 15 OAM channels, and arranges the demultiplexed channels at equally-spaced positions over the same circle in far-field. The second DOE extends the OAM set to {−3, −2, −1, 0, +1, +2, 3} and performs sorting over a total of 21 channels, arranged over three concentric circles in far-field. The limitation to three beam sizes is not fundamental. Additional annuli could be designed extending the DOE size, otherwise the beam ring width could be reduced in order to accommodate more DOE rings on the same area. Likewise, the OAM set can be further extended and OAM values are not required to be necessarily consecutive. In particular, the choice of non-consecutive OAM values could remarkably diminish channel cross-talk. As a matter of fact, a generic approach to minimize cross-talk is to increase the separation between channels (see Supplementary Figure 2). The exploitation of perfect vortices is essential in order to control radius and width of the generated ring-shaped beam and, in particular, to make it independent of its OAM content.

## Methods

### Numerical simulations

A custom MATLAB code was implemented in order to compute the DOE phase pattern. The designed phase masks loaded onto SLM for perfect vortex generation were Bitmap figures generated by a custom MATLAB routine.

### Electron beam lithography

All 3D multilevel structures have been fabricated in a 2 μm thick polymethylmethacrylate (PMMA) resist with a molecular weight of 950 k (kg/mol), spin-coated on a 1.1 mm thick ITO coated soda lime float glass substrate and prebaked for 10 min at 180 °C on a hot plate. For the gray-scale lithography step, a dose-depth correlation (contrast curve) was used. Contact profilometry was performed to determine the remaining resist heights. Dose-to-clear value (complete removal of PMMA) was found to be 566 μC/cm^2^. DOE patterns were written with a JBX-6300FS JEOL EBL machine, 12 MHz, 5 nm resolution, working at 100 keV with a current of 100 pA. The presence of the ITO layer (resistivity 8–12 Ω) was necessary in order to ensure a good discharge of the sample during electron beam lithography. A dose correction for the compensation of proximity effects has been applied. This compensation is required both to match layout depth with the fabricated relief and to obtain a good shape definition, especially in correspondence of the phase steps. Exposed samples were developed under slight agitation in a temperature-controlled developer bath for 60 s. Deionized water: isopropyl alcohol (IPA) 3:7 was found to be the most suitable developer, giving optimized sensitivity and contrast characteristics as well as a minimized pattern surface roughness at 20 °C. After development, the samples were gently rinsed in deionized water and blow dried using nitrogen flux. Different techniques have been used in order to assess sample quality: tapping-mode atomic force microscopy (AFM), optical microscopy and scanning electron microscopy (SEM).

### Optical characterization

The characterization setup was designed and assembled on an optical table with gimbal piston isolators. The Gaussian beam is emitted by a HeNe laser source (HNR008R, Thorlabs, *λ* = 632.8 nm, waist *w*_0_ = 240 μm, power 0.8 mW). The beam is polarized (LPVISE100-A, Thorlabs) and illuminates the display of a reflective liquid-crystal-on-silicon (LCoS) spatial light modulator (PLUTO-NIR-010-A, Holoeye, 1920 × 1080 pixels, 8 μm pixel size, 8-bit depth), optimized for use at 632.8 nm. Then the reflected beam is collimated with a first lens of focal length *f*_1_ = 25 cm and a beam-splitter is used to analyze the field profile and its OAM content at the same time with two different acquisition cameras. The field profile is collected with a CMOS camera (DCC1545M, Thorlabs, 1280 × 1024 pixels, 5.2 μm pixel size, monochrome, 8-bit depth). The sample is fixed on a vertical *XY* translation mount with micrometric drives (ST1XY-S/M, Thorlabs, travel 2.5 mm, resolution 10 μm), and the far-field is collected with a second CCD camera (1500M-GE, Thorlabs, 1392 × 1040 pxls, 6.45 μm pixel size, monochrome, 12-bit depth) placed at the back-focal plane of a lens with focal length *f*_2_ = 10 cm.

## Additional Information

**How to cite this article**: Ruffato, G. *et al*. Diffractive optics for combined spatial- and mode-division demultiplexing of optical vortices: design, fabrication and optical characterization. *Sci. Rep.* **6**, 24760; doi: 10.1038/srep24760 (2016).

## Supplementary Material

Supplementary Information
