# Supplementary material for: Diffractive optics for combined spatial- and mode- division demultiplexing of optical vortices: design, fabrication and optical characterization
Source: Sci Rep. 2016 Apr 20;6:24760. doi: 10.1038/srep24760 (PMC4837364; doi:10.1038/srep24760)
Supplement: Supplementary Information [file srep24760-s1.pdf]

Supplementary information

Diffractive optics for combined spatial- and mode-division demultiplexing of optical vortices: design, fabrication and optical characterization

Gianluca Ruffato[1,2*], Michele Massari[1,2], and Filippo Romanato[1,2,3]

[1]Department of Physics and Astronomy ‘G. Galilei’ ,University of Padova, via Marzolo 8,35131 Padova, Italy

[2]LaNN, Laboratory for Nanofabrication of Nanodevices, EcamRicert, C.so Stati Uniti 4, 35127 Padova, Italy.

[3]CNR-INFM TASC IOM National Laboratory, S.S. 14 Km 163.5, 34012 Basovizza, Trieste, Italy

[*]Corresponding author: gianluca.ruffato@unipd.it

S1. AFM ANALYSIS

Atomic Force Atomic (AFM) microscopy was performed in tapping-mode configuration. For a 8-level phase-only DOE in PMMA, working in transmission at λ=632.8 nm, nominal depth values are: d_1=0 nm, d_2=161.8 nm, d_3=323.5 nm, d_4=485.3 nm, d_5=647.0 nm, d_6=808.8 nm, d_7=970.6 nm, d_8=1132.3 nm. In Supplementary figure 1, a 3D AFM reconstruction is reported for a small area of 20 x 20 μm^2. Experimental values are compared with the nominal ones exhibiting a remarkable accordance within the experimental errors, estimated by considering surface roughness. Roughness root-mean-square (RMS) increases from 4 nm to 32 nm, for the lowest and highest depth level respectively.

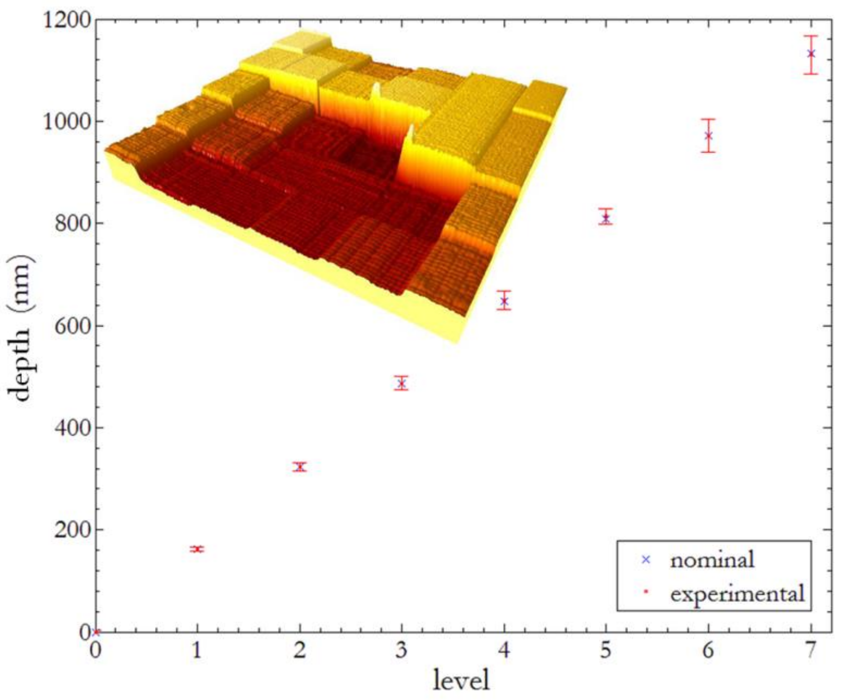

Supplementary Figure 1. AFM analysis of 20 x 20 μm^2 (spanning about 6 x 6 pixels) zone of a 8-level DOE performing OAM-MDM in the range {-2,-1,0,+1,+2}. Comparison between experimental (dot with error bars) and nominal (cross) heights. Roughness is lower than 3 % for each thickness level.

S2. CROSS-TALK REDUCTION BY INCREASING OAM CHANNEL SEPARATION

A generic approach in order to minimize inter-channel cross-talk consists in increasing OAM separation between channels. In the following we consider the optical response of three different DOEs performing OAM-MDM in a set of 7 vortices with increasing OAM separation $\Delta\ell$=1, 2, 3, respectively in the sets {-3, -2, -1, 0, +1, +2, +3}, {-6, -4, -2, 0, +2, +4, +6}, {-9, -6, -3, 0, +3, +6, +9}, working wavelength λ = 632.8 nm. DOE geometry is the same: inner radius 300 μm, outer radius 500 μm. Pixel size: 4 x 4 μm^2. In far-field, signal peaks are distributed over the same circle and equally spaced.

For each DOE we calculated the experimental total efficiency and channel cross-talk (see Supplementary figure 2). For increasing channel separation, there is a dramatic improvement in cross-talk values. As a matter of fact, since the main spurious contributions originate from the nearest-neighbours OAM values, increasing $\Delta\ell$ reduces the overlap between the signal peak and the surrounding doughnuts related to different ℓ channels. For instance, we report the cross-talk of the channels corresponding to ℓ=+1, ℓ=+2, ℓ=+3, for the three DOEs respectively:

$$XT_{\ell=+1}^{\Delta\ell=1} = 10 \cdot \log_{10} \frac{I_{+1,ALL\backslash\{+1\}}}{I_{+1,ALL}} = -5.8$$

$$XT_{\ell=+2}^{\Delta\ell=2} = 10 \cdot \log_{10} \frac{I_{+2,ALL\backslash\{+2\}}}{I_{+2,ALL}} = -14.5$$

$$XT_{\ell=+3}^{\Delta\ell=3} = 10 \cdot \log_{10} \frac{I_{+3,ALL\backslash\{+3\}}}{I_{+3,ALL}} = -22.2 \qquad (1)$$

where $I_{\ell^*,ALL}$ is the signal in correspondence of channel ℓ^* when all input OAM signals in the set $\{\ell_i\}$ are on, ℓ^* included, while $I_{ALL\backslash(\ell^*)}$ is the signal at channel ℓ^* when the input channel ℓ^* is off. It follows that channel efficiency is remarkably improved as well, as the next values show for the previously selected channels:

$$\eta_{(+1,+1)}^{\Delta\ell=1} = 0.79$$

$$\eta_{(+2,+2)}^{\Delta\ell=2} = 0.95$$

$$\eta_{(+3,+3)}^{\Delta\ell=3} = 0.99 \qquad (2)$$

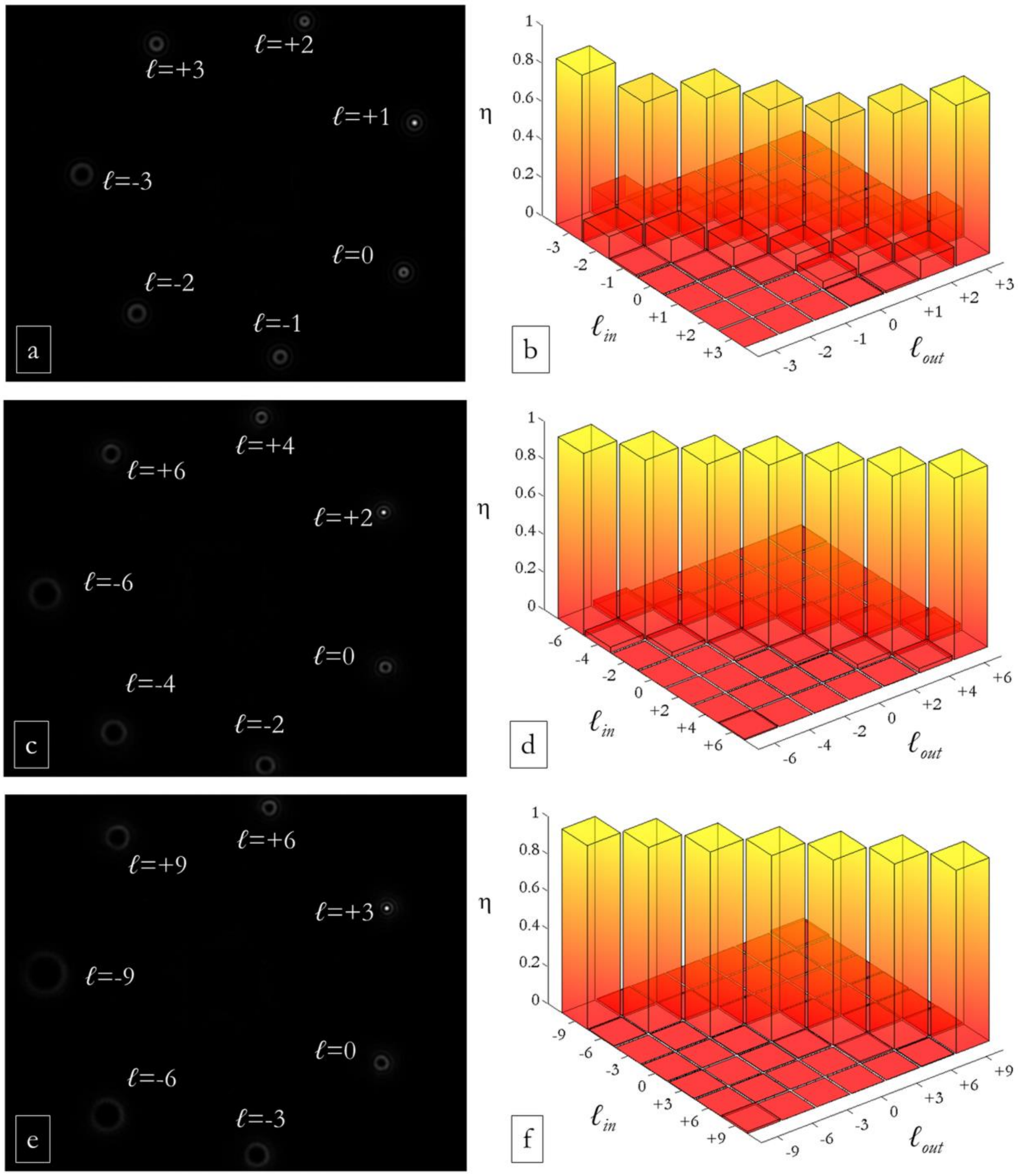

Supplementary figure 2. Experimental optical response for DOEs performing OAM-MDM over three different OAM sets: {-3, …, +3}, step $\Delta\ell$=1 (a,b); {-6, …, +6}, step $\Delta\ell$=2 (c,d); {-9, …, +9}, step $\Delta\ell$=3 (e,f). (a, c, e) Experimental output for input modes OAM_{+1}, OAM_{+2} and OAM_{+3} for the three DOEs respectively. (b, d, f) Normalized intensities in all detector regions for perfect vortex input modes, experimental data. For each channel, detection regions have the same size and are chosen so that they cover the intensity peak area. Intensities are normalized to the total collected energy.
